# Unsupervised background-constrained tank segmentation of infrared images in complex background based on the Otsu method

**DOI:** 10.1186/s40064-016-3094-4

**Published:** 2016-08-24

**Authors:** Yulong Zhou, Min Gao, Dan Fang, Baoquan Zhang

**Affiliations:** 1 Electronic Engineering Department, Shijiazhuang Mechanical Engineering College, Heping Road, Shijiazhuang City, 050003 China; 2 66393 Postdoctoral Science Research Workstation, Qiyi Road, Hebei Baoding City, 071000 China; 3 State Grid Hebei Electronic Power Company Maintenance Branch, XinHua Region, Zhongsheng Road No. 66, Shijiazhuang, 050071 China

**Keywords:** Infrared images in complex background, Tank segmentation, The Otsu method, Threshold analysis, Background constraint

## Abstract

In an effort to implement fast and effective tank segmentation from infrared images in complex background, the threshold of the maximum between-class variance method (i.e., the Otsu method) is analyzed and the working mechanism of the Otsu method is discussed. Subsequently, a fast and effective method for tank segmentation from infrared images in complex background is proposed based on the Otsu method via constraining the complex background of the image. Considering the complexity of background, the original image is firstly divided into three classes of target region, middle background and lower background via maximizing the sum of their between-class variances. Then, the unsupervised background constraint is implemented based on the within-class variance of target region and hence the original image can be simplified. Finally, the Otsu method is applied to simplified image for threshold selection. Experimental results on a variety of tank infrared images (880 × 480 pixels) in complex background demonstrate that the proposed method enjoys better segmentation performance and even could be comparative with the manual segmentation in segmented results. In addition, its average running time is only 9.22 ms, implying the new method with good performance in real time processing.

## Background

In military fields, forward-looking infrared (FLIR) systems of long wave infrared (LWIR) are widely used to improve the night fighting capability, such as missile guidance and visual supervision systems. Accordingly, infrared image processing techniques, such as infrared image segmentation, target recognition, target tracking and so on, are paid much more and more attentions by lots of researchers and have become one of hot spots in present research (Fan et al. 2011).

During infrared image processing techniques, infrared image segmentation is a basic preprocessing step in infrared image analysis and computer vision (Sen and Pal 2010; Huang and Wang 2009). It intends to separate an object from a background based on some pertinent characteristics such as gray level, gray gradient, texture and location (Tao et al. 2008). Up to now, there have been many effective segmentation algorithms proposed by researchers. However, when the background of an infrared image is complex, the conventional algorithms will tend to be poor and even fail in segmentation. To solve this problem, lots of novel algorithms are proposed to improve segmentation accuracy. But the performance improvement of the approach is always at the cost of huge increase of computing time, such as the 2-D Otsu method, the 2-D maximum entropy method and so on, resulting that the improved method hardly meet the requirement of the real time processing (Guo et al. 2006).

Among the existing segmentation techniques, thresholding is one of the most popular approaches in terms of simplicity, robustness and accuracy (Li et al. 2011). Implicit assumption in image thresholding is that target and background have distinctive gray levels. Thresholding serves a variety of applications, such as biomedical image analysis, character identification and industrial inspection. Many thresholding approaches and their improvements have been developed over the last few years (Long et al. 2012; Liu and Jin 2013; Wu et al. 2010; Li and Tian 2009; Qiao et al. 2013). The most popular thresholding algorithms include the maximum between-class variance method (namely the Otsu algorithm), the entropy based thresholding algorithm, the minimum error method, the paragenetic matrix method, the moments method, the probability relaxation method and so on. Of these methods, the maximum between-class variance method proposed by Otsu was widely used for its simple calculation and good self-adaptive ability (Hu et al. 2010; Zhang et al. 2006). It is based on the single order statistical characteristics of gray histogram, and hence possesses rapid calculation and real-time processing advantages. But to different kinds of infrared images, the Otsu algorithm can’t always produce the results we wanted. Especially when the image background is very complex and the ratio of target to background is very small, the Otsu method will be poor and even fail to segment the area of our interest from the background. So in this paper, with fully considering the real-time processing requirement of infrared imaging guidance system, the threshold obtained by Otsu method will be examined for tank infrared images in complex background and the essential reason of the algorithm failing in tank image segmentation will be found out. Then the improved method will be proposed to solve the exposed problem.

## The Otsu method and its threshold analysis

### The Otsu method

The maximum between-class variance method, namely the Otsu method, was proposed by Otsu, which is based on single dimension gray histogram of the image and makes maximizing the variance between classes of background and target regions as threshold selection criterion. Suppose that the image pixels are divided into two classes of $C_{0}$ and $C_{1}$ by gray value *T*. Let $C_{0} = [1, \ldots ,T]$ and $C_{1} = [T + 1, \ldots ,L - 1]$. Let $P_{0} (T)$ and $P_{1} (T)$ respectively denote the probabilities of $C_{0}$ and $C_{1}$. Correspondingly, $\mu_{0} (T)$ and $\mu_{1} (T)$ are the gray means, and $\sigma_{0}^{2} (T)$ and $\sigma_{1}^{2} (T)$ are the variances of two classes, respectively. Then some important calculations are as follows (Otsu 1979).

The mean of the image $\mu$ is obtained by

1 $$\mu = P_{0} (T) \, \mu_{0} (T) + P_{1} (T) \, \mu_{1} (T).$$

The between-class variance (BCV) $\sigma_{b}^{2} (T)$ is obtained via

2 $$\sigma_{b}^{2} (T) = P_{0} (T)( \, \mu_{0} (T) - \mu )^{2} + P_{1} (T)( \, \mu_{1} (T) - \mu )^{2} .$$

The sum of within-class variances (WCVs) of two classes $\sigma_{\omega }^{2} (T)$ is given by

3 $$\sigma_{\omega }^{2} (T) = P_{0} (T)\sigma_{0}^{2} (T) + P_{1} (T)\sigma_{1}^{2} (T).$$

Let $T^{*}$ denote the threshold obtained by maximizing BCV of the image, then

4 $$\sigma_{b}^{2} (T^{*} ) = \mathop {\hbox{max} }\limits_{1 \le T < L} \sigma_{b}^{2} (T).$$

Let $T^{{{\prime }*}}$ denote the threshold by minimizing the sum of WCVs of the image, then

5 $$\sigma_{\omega }^{2} (T^{{{\prime }*}} ) = \mathop {\hbox{min} }\limits_{1 \le T < L} \sigma_{\omega }^{2} (T).$$

Otsu pointed out that the two selection criteria of Eqs. (4) and (5) are equivalent, and hence they will possess the same threshold value (Otsu 1979).

### Threshold analysis of the Otsu method

**Fig. 1:**
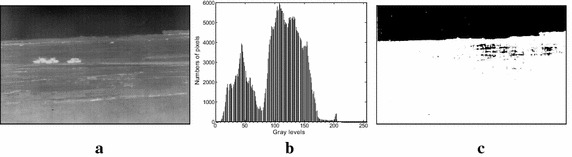
Original infrared image, its histogram and segmented result by the Otsu method: **a** three tanks infrared image, **b** histogram, **c** segmented result by the Otsu method

In this paper, a large number of infrared tank images produced by the infrared imaging guidance system are adopted to carry out the experiments with the Otsu method. Due to the limited space, and for the sake of description convenience, take the three tanks infrared image with complex background in Fig. 1a as an example. It is of 880 × 480 pixels and 8-bit (i.e., 256 gray levels). In the image, the average ratio of target to background for three tanks is only 1.1 %, which indicates that they are small targets. In addition, there are lots of dark and bright regions in the image, which makes the image more complex. Figure 1b shows the gray histogram of Fig. 1a. From the figure, it is found that the gray level range of Fig. 1a is very big, indicating that the original image is rich in varieties of gray levels.

For the sake of description convenience, the manual segmentation result is regarded as the optimal result for infrared image, and the corresponding threshold will be treated as the optimal threshold. Figure 1c is the segmented result by the Otsu method, which shows that there are lots of background pixels in segmented image and the targets completely overlap with the background. As a result, the visual effect of the segmented result is poor, implying the failure of Otsu method in segmenting the area of interest from infrared images in complex background.

**Fig. 2:**
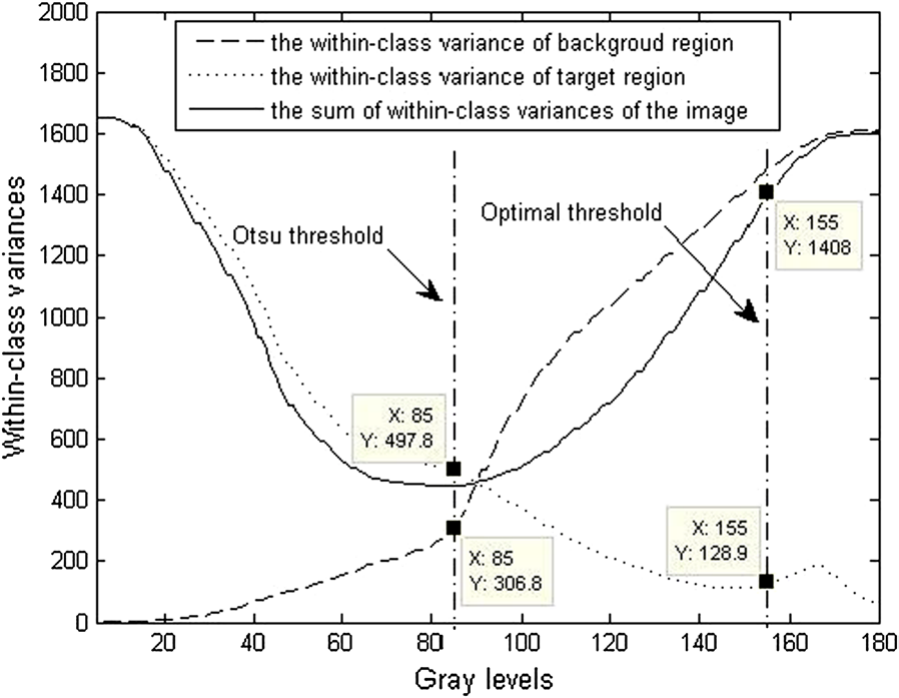
Curves of the WCVs for Fig. 1a

Otsu pointed out that the threshold selection criteria of Eqs. (4) and (5) are equivalent. In order to find out the underlying reason for influence on the threshold, the selection criterion of the minimum within-class variance (MWCV) based on Eq. (5) is analyzed. According to Eq. (5), when the sum of WCVs gets to its minimum, the corresponding gray level will be the value of the threshold obtained by the Otsu method (i.e., the Otsu threshold). In addition, together with Eq. (3), one can find that the Otsu threshold has close relations with the WCVs of target region and background. As a result, the curves of WCV changed as gray levels for Fig. 1a are displayed in Fig. 2. From the figure, it can be observed that, as gray level increasing, the WCV of the background region becomes bigger and that of the target region gets smaller, gradually; the sum of WCVs of the image has a minimum value, where the corresponding gray level is the Otsu threshold. The Otsu and the optimal thresholds are marked respectively with dotted lines in Fig. 2, and it is found that, the Otsu threshold is much smaller than the optimal threshold and the Otsu threshold place is extremely close to the point where the two classes have the same WCV value, indicating the small difference between the WCVs of two classes; by contrast, the place of the optimal threshold is far away from that point, implying that the difference between the WCVs is big (as shown in Fig. 2, the WCV of background region is about 11 times bigger than that of the target region). As a result, it can be concluded that just because of the big difference between the WCVs of the background and the target regions, the Otsu threshold is made to deviate from the optimum value (the bias is towards the component with bigger WCV), resulting that the Otsu method fails in segmenting the area of interest from the image background. This conclusion is consistent with the theoretical analysis result by Xu et al. (2009).

Furthermore, the working mechanism of the Otsu method can also be presented. The small difference between WCVs of the background and the target regions is necessary for the Otsu method to produce satisfying segmented results. Therefore, to improve the Otsu method segmentation ability for the small target infrared images with complex background, the WCV of the background region must be lowered to get close to that of the target region. Subsequently, a new scheme to eliminate the negative influence of the complex background on the Otsu method has been designed and hence an unsupervised background constrained thresholding method is proposed based on the Otsu method in the following content.

## Unsupervised background constrained thresholding method based on the Otsu method

A new scheme will be tested to eliminate the negative influence of the complex background on the Otsu method in this section so as to improve the performance of the Otsu method. The scheme first divides the infrared image into three classes of target region, middle background and lower background via the way of maximizing the between-class variance (BCV)s of three classes, then finds the lowest bound of the gray levels for the infrared image based on the within-class variance (WCV) of the target region and hence simplifies the original infrared image by background constraint with the gray level range, finally applies the Otsu method to the simplified image, thus forming an unsupervised background constrained thresholding algorithm based on the Otsu method with better segmentation performance.

### The classes division of an infrared image with complex background

Based on the analysis result in “Threshold analysis of the Otsu method”, one can conclude that the Otsu method will not get satisfactory segmentation until the WCV of the background gets close to that of the target region. For the infrared images in complex background, the range of gray levels is very big. That is to say, the background is rich in varieties of gray levels including not only the lower gray levels, but also the higher gray levels. So it will not be reasonable to simply divide the image into two classes comprised of background and target regions. Otherwise, there will be more background pixels to be misclassified into the target region, resulting that the Otsu method fails in segmentation. So if the infrared image with complex background is divided into three classes comprised of the target region, the middle background and the lower background by the Otsu method via the image statistical characteristics, it will be more reasonable to reflect the reality of the image and hence there will be more background pixels classified into the class of middle background and less ones misclassified into the class of target region.

In all of our discussions, we assume that the target region pixels have higher gray levels than the background ones. In order to divide the image into three classes, two gray levels should be chosen as the lower bound for the target region and the upper bound for the lower background by maximizing the sum of three classes’ BCVs. After finding the lower and upper bounds, gray level ranges of the target region, the middle background and the lower background are accordingly determined, respectively. The detailed steps are as follows:

1. Compute the mean of the image $\mu$ with Eq. (1).
2. Define two gray levels
  6 $$\left\{ {\begin{array}{*{20}c} {T_{1} = \mu - i} \\ {T_{2} = \mu + i} \\ \end{array} } \right.\quad i \ge 1\;{\text{and}}\;i \le \hbox{min} (\mu ,255 - \mu ),$$
  where *i* is a parameter and its value can be automatically determined by maximizing the proposed criterion in Eq. (11). *T*_1_ and *T*_2_ are the upper and lower bounds for lower background and target region, respectively.
3. Find a reasonable value for parameter *i* via the statistical characteristics of the image. Determining the upper and lower bounds needs choosing a reasonable value for parameter *i*, which in turn involves a statistical criterion to be defined.

Suppose that the image *I* is divided into three classes by the two gray levels *t*_1_ and *t*_2_, where *t*_1_ < *t*_2_, with three classes represented by $C_{l}$, $C_{m}$ and $C_{o}$, where $C_{l}$ is the lower background class with gray levels $[0, \ldots t_{1} ]$, $C_{m}$ the middle background class with levels $[t_{1} + 1, \ldots ,t_{2} ]$ and $C_{o}$ the objet region class with levels $[t_{2} + 1, \ldots ,L - 1]$. The mean of two classes of $C_{l}$ and $C_{m}$ can be obtained via

7 $$\mu_{lm} = \frac{1}{{N_{lm} }}\sum\limits_{i = 0}^{{t_{2} }} {in_{i} } ,$$

and that of $C_{m}$ and $C_{o}$ can be given by

8 $$\mu_{mo} = \frac{1}{{N_{mo} }}\sum\limits_{{i = t_{1} }}^{L - 1} {in_{i} } ,$$

where $N_{lm}$ is the total number of pixels in $C_{l}$ and $C_{m}$, and $N_{mo}$ is that of pixels in $C_{m}$ and $C_{o}$. Assume that $\sigma_{lm}^{2}$ denotes the BCV between the classes of $C_{l}$, $C_{m}$, and $\sigma_{mo}^{2}$ denotes that between $C_{m}$ and $C_{o}$, then they can be calculated respectively as follows:

9 $$\sigma_{lm}^{2} = \frac{{N_{l} }}{{N_{lm} }}( \mu_{l} - \mu_{lm} )^{2} + \frac{{N_{m} }}{{N_{lm} }}(\mu_{m} - \mu_{lm} )^{2} ,$$

10 $$\sigma_{mo}^{2} = \frac{{N_{m} }}{{N_{mo} }}(\mu_{m} - \mu_{mo} )^{2} + \frac{{N_{o} }}{{N_{mo} }}(\mu_{o} - \mu_{mo} )^{2} ,$$

where $\mu_{l}$, $\mu_{m}$ and $\mu_{o}$ are the mean of each class respectively.

Based on Eqs. (9) and (10), the statistical criterion could be defined as

11 $$\sigma_{S}^{2} = P_{lm} \sigma_{lm}^{2} + P_{mo} \sigma_{mo}^{2} ,$$

where $P_{lm}$, $P_{mo}$ are the weights balancing the contributions of the two terms and they can be given by

12 $$P_{lm} = \frac{{N_{lm} }}{{N_{S} }},$$

13 $$P_{mo} = \frac{{N_{mo} }}{{N_{S} }},$$

where $N_{S}$ is the number of all pixels in the original image.

**Fig. 3:**
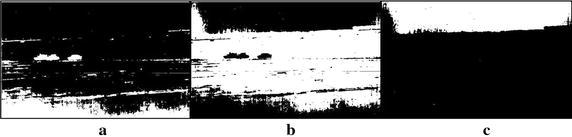
Three classes divided by the proposed statistical criterion (*i* = 37, *T*_1_ = 65, *T*_2_ = 139): **a** target region, **b** ground background (*middle background*), **c** sky background (*lower background*)

The criterion stands for the sum of BCVs during classes of $C_{o}$, $C_{m}$ and $C_{l}$. The BCV is a common statistical method reflecting degree of differences between classes (Kurz and Benteftifa 1997). In other words, it in turn reflects the degree of similarities within the class. Hence, $\sigma_{S}^{2}$ could represent the between-class differences during the target region, middle background and lower background to some extent. The bigger the $\sigma_{S}^{2}$ is, the higher the differences during classes are, also implying the higher the similarities within the class. Select the value of parameter *i* from 1 to $\hbox{min} (\mu ,255 - \mu )$ to compute $\sigma_{S}^{2}$ with Eq. (11) until it reaches its maximum, the value for parameter *i* can be determined and hence the upper and lower bounds, *T*_1_ and *T*_2_, can be calculated by Eq. (6). Take the infrared image with three tanks in Fig. 1a for an example. The figure shows that the original infrared image consists of targets, ground background and sky background, with the higher gray levels, middle levels and lower levels, correspondingly. After computation by statistical criterion of Eq. (11), the value of the parameter *i* is automatically set to 37 and the upper and lower bounds are 65 and 139 respectively. Its target region, middle background and lower background are displayed in Fig. 3a–c, where the bright pixels are our focuses. In the figure, it can be observed that the target region, the ground and sky backgrounds are better separated from the original infrared image respectively. However, concerning the target region, there are still many background pixels left adhering to the targets in target region as shown in Fig. 3a. So it still needs extra steps to make the targets completely segmented. For example, background constraint and image normalization need to be implemented.

### Background constraint based on the within-class variance of the target region

From the analysis results in “The Otsu method and its threshold analysis”, one can concluded that the Otsu method could not have better performance in image segmentation until the WCV of background gets close to that of the target region. In this section, the target region obtained from “The classes division of an infrared image with complex background” is treated as the optimal approximately. The background constraint will be implemented based on the WCV of the target region. The detailed process is as follows:

1. Calculate the mean of target region by
  14 $$\mu_{o} = \frac{1}{{N_{o} }}\sum\limits_{{i = T_{2} + 1}}^{L - 1} {in_{i} } ,$$
  where *T*_2_ is the lower bound of gray levels for target region.
2. Calculate the WCV of target region via
  15 $$\sigma_{o}^{2} (T) = \frac{1}{{N_{o} }}\sum\limits_{{i = T_{2} }}^{L - 1} {(i - \mu_{o} )^{2} } n_{i} .$$
3. Find the lower bound of gray levels for background. Assume that the lower bound of gray levels of background is denoted by *T*_*c*_, where *T*_*c*_ < *T*_2_. Let *T*_*c*_ decrease from *T*_2_ and thus the gray levels $[T_{c} , \ldots ,T_{2} ]$ are treated as the levels of background. Decrease *T*_*c*_ until the WCV of background equals to that of target region, and the lower bound will be determined.
4. Implement the background constraint via the following way:
  16 $$f_{c} (i,j) = \left\{ {\begin{array}{*{20}l} 0 \hfill & {{\text{if}}\;f(i,j) < T_{c} } \hfill \\ {f(i,j)} \hfill & {{\text{if}}\;f(i,j) \ge T_{c} } \hfill \\ \end{array} } \right.,$$
  where $f_{{}} (i,j)$ and $f_{c} (i,j)$ are the gray levels at pixel (*i*, *j*) of the original infrared image and the constrained form, respectively.

**Fig. 4:**
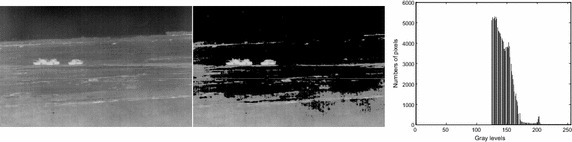
Original image and its constrained results: **a** original, **b** simplified image after background constraint, **c** histogram after background constraint

The background constraint weakens the gray level changes in complex background, thus simplifying the original infrared image. This is beneficial to subsequent image segmentation. Take background constrained form Fig. 4b for Fig. 1a as an example. Their histograms are displayed in Fig. 1b and Fig. 4c respectively. From these figures, it can be observed that gray level changes of background are weakened dramatically and the background constrained image becomes much simpler than the original.

### Detailed steps of the proposed method

After the complex background constraint, the Otsu method can be adopted to find the optimal threshold by statistical characteristics of the simplified infrared image. The whole process of the proposed method is as follows:

1. Divide infrared image into three classes of target region, middle background and lower background via maximazing the statistical criterion of Eq. (11).
2. Find the lower bound of background *T*_*c*_. Treat the target region as the optimal one approximately and select the value from *T*_2_ to 1 to calculate the within-class variance of background until it equal to the within-class variance of target region. Then the value of *T*_*c*_ is determined.
3. Implement the background constraint via Eq. (16) and thus simplify the infrared image.
4. Apply the Otsu method to segment the simplified infrared image.

## Experiments and analysis

**Fig. 5:**
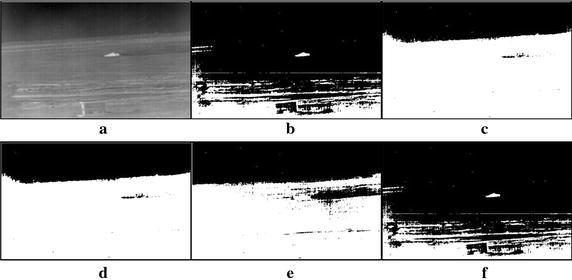
Thresholding results of single tank infrared image: **a** original infrared image (ratio = 0.2 %), **b** manual segmentation, **c** standard Otsu method, **d** 2-D Otsu method, **e** 2-D maximum entropy, **f** this paper method

**Fig. 6:**
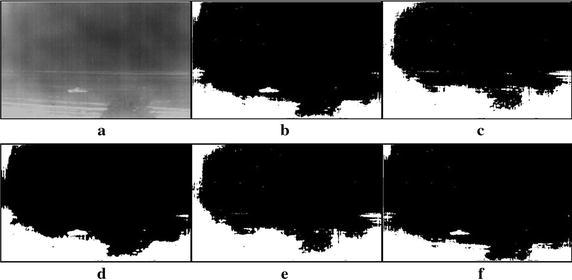
Thresholding results of single tank infrared image: **a** original infrared image (ratio = 0.3 %), **b** manual segmentation, **c** standard Otsu method, **d** 2-D Otsu method, **e** 2-D maximum entropy, **f** this paper method

**Fig. 7:**
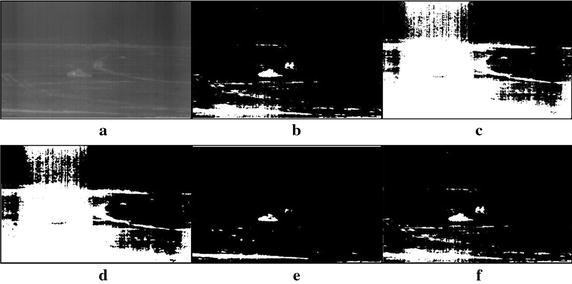
Thresholding results of single tank infrared image: **a** original infrared image (ratio = 0.5 %), **b** manual segmentation, **c** standard Otsu method, **d** 2-D Otsu method, **e** 2-D maximum entropy, **f** this paper method

**Fig. 8:**
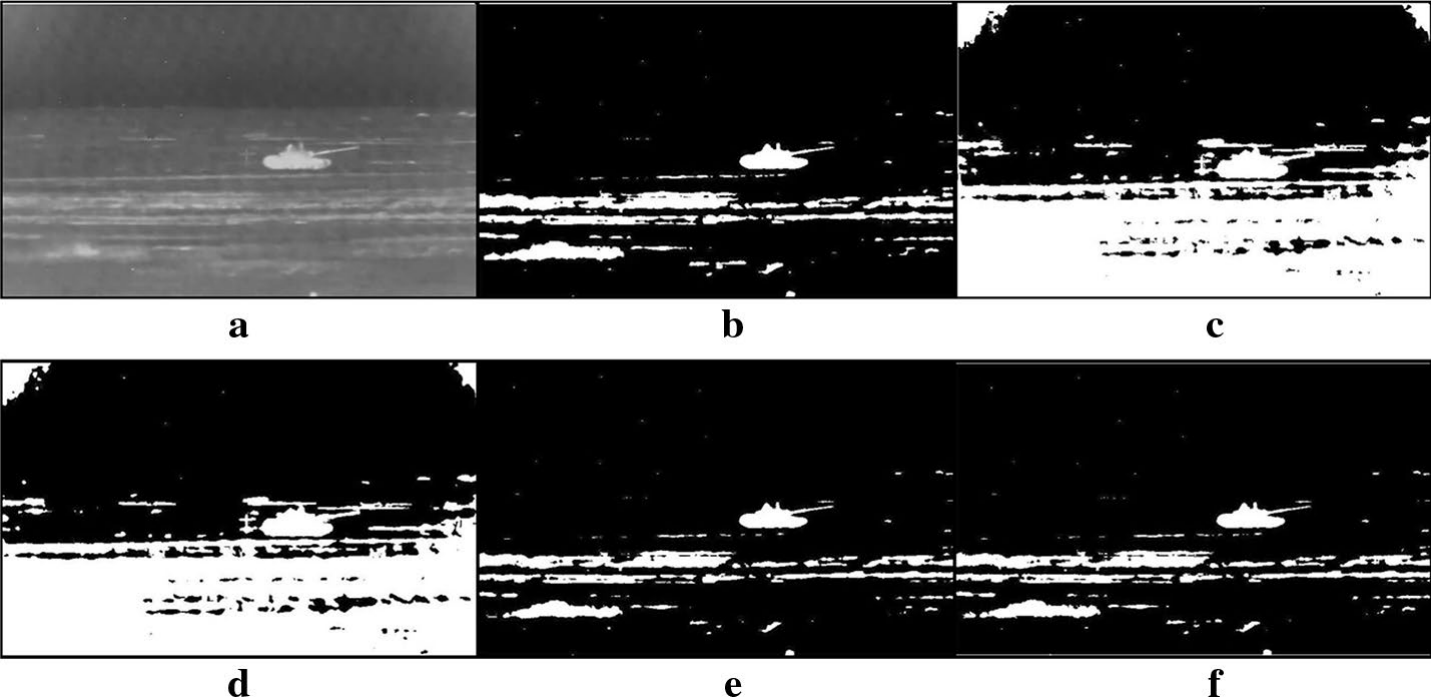
Thresholding results of single tank infrared image: **a** original infrared image (ratio = 0.8 %), **b** manual segmentation, **c** standard Otsu method, **d** 2-D Otsu method, **e** 2-D maximum entropy, **f** this paper method

**Fig. 9:**
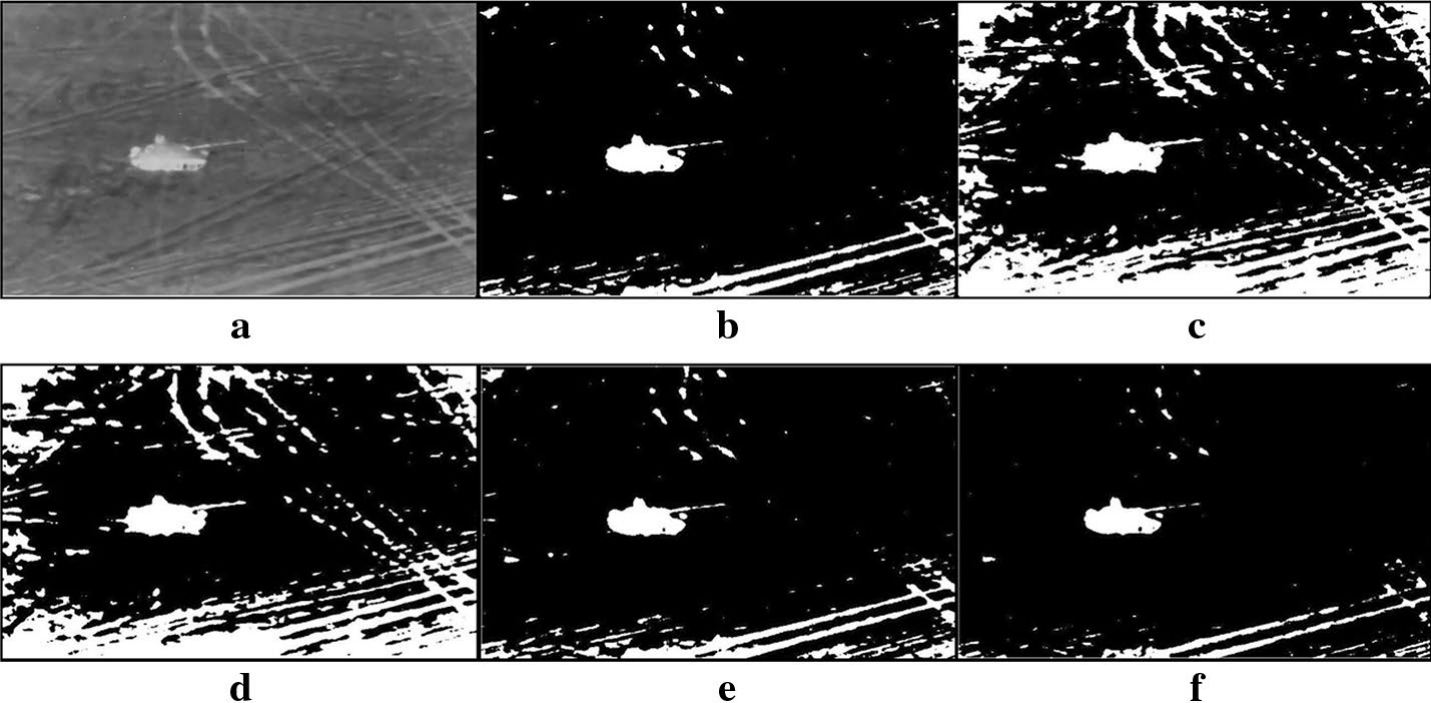
Thresholding results of single tank infrared image: **a** original infrared image (ratio = 1.3 %), **b** manual segmentation, **c** standard Otsu method, **d** 2-D Otsu method, **e** 2-D maximum entropy, **f** this paper method

**Fig. 10:**
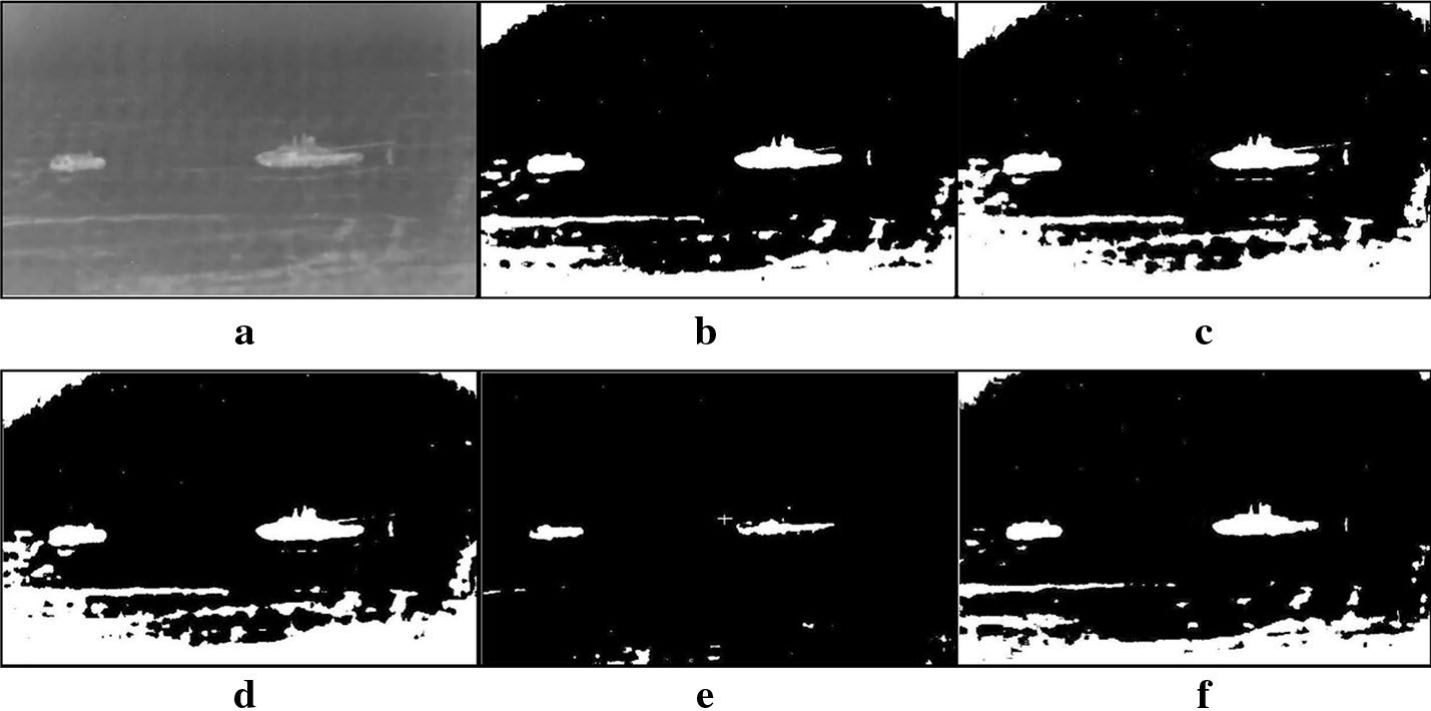
Thresholding results of two targets infrared image: **a** original infrared image, **b** manual segmentation, **c** standard Otsu method, **d** 2-D Otsu method, **e** 2-D maximum entropy, **f** this paper method

**Fig. 11:**
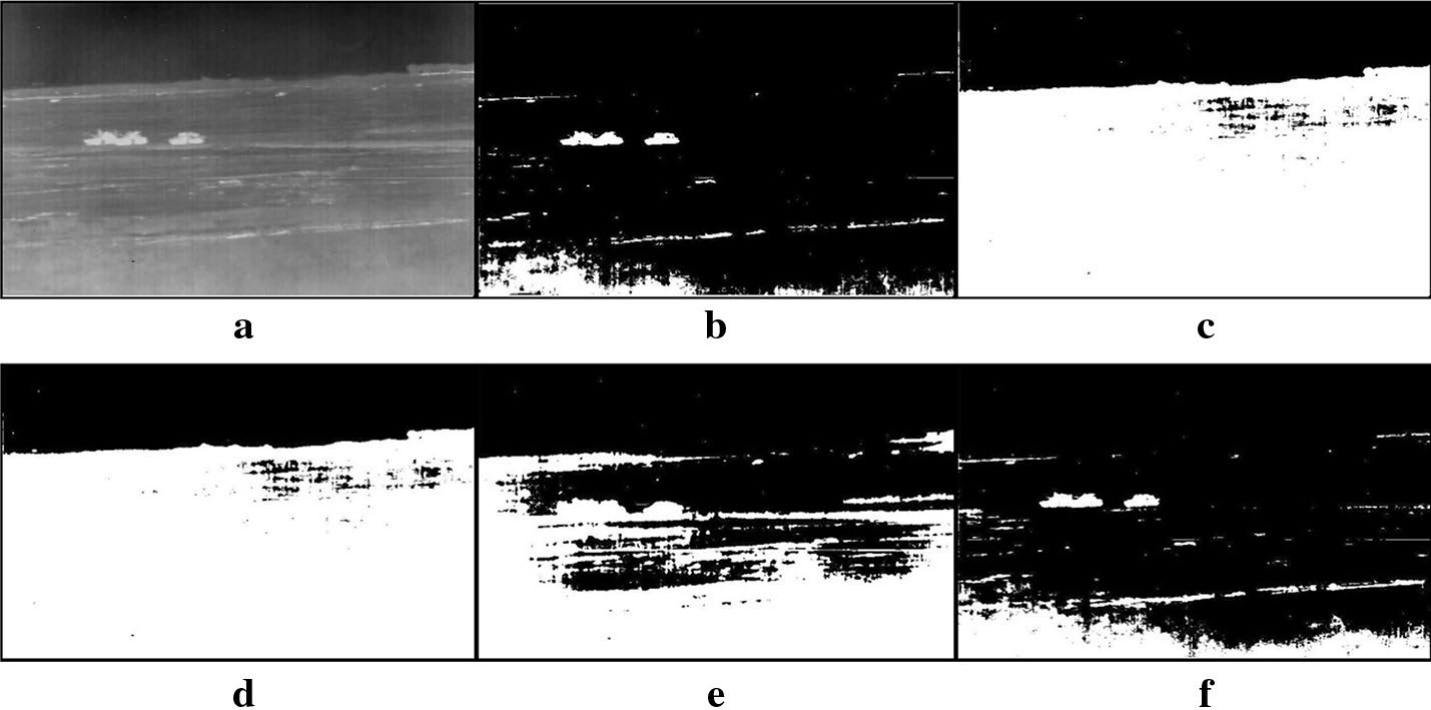
Thresholding results of three tanks infrared image: **a** original infrared image, **b** manual segmentation, **c** standard Otsu method, **d** 2-D Otsu method, **e** 2-D maximum entropy, **f** this paper method

**Fig. 12:**
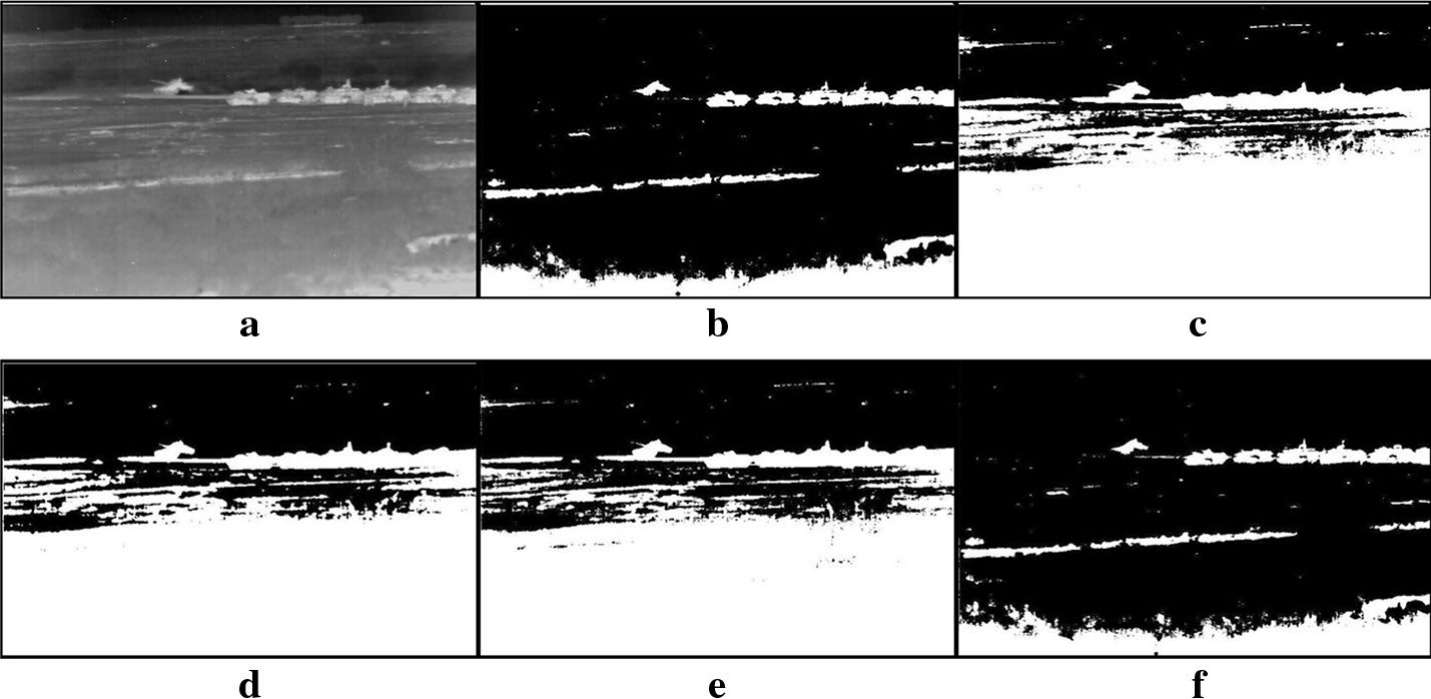
Thresholding results of multiple tanks infrared image: **a** original infrared image, **b** manual segmentation, **c** standard Otsu method, **d** 2-D Otsu method, **e** 2-D maximum entropy, **f** this paper method

A large number of tank infrared images of real world have been used to take the experiments so that the performance of the proposed method can be evaluated. All experiments are carried out based on the MATLAB 7.1 version on a notebook PC with 2.2 G Intel Core 2 Duo CPU and 2 G RAM. Due to the limited space, seven tank infrared images have been chosen as testing samples, which are displayed in Figs. 5, 6, 7, 8, 9, 10, 11 and 12. These images fall into two groups, the first is for single target images with different ratios of target to image, and the second for multiple targets images. All the original infrared images are of 800 × 600 pixels and with complex background in different extent. The results produced by the proposed method were compared with those obtained by different methods, i.e., the standard Otsu method, the 2-D Otsu method and the 2-D maximum entropy method. Of them, unlike that the standard Otsu method is based on single dimension gray histogram, the latter two methods are both based on two dimension histogram and hence their segmentation performance are improved comparing with the single dimension histogram based ones. However, their consuming times are also increased dramatically.

### Segmentation results comparison of different methods

For description convenience, the manual segmentation results are treated as the optimal ones and displayed in the figures from Figs. 5b, 6b, 7b, 8b, 9b, 10b, 11b and 12b. The segmented results by different methods are also given in Figs. 5, 6, 7, 8, 9, 10, 11 and 12, respectively. From them, it can be observed that, due to the complex background, the segmented results by the standard Otsu method are the worst in visual effect and those by both the 2-D Otsu method and the 2-D maximum entropy method are slightly better. That is to say, there are still more images not well segmented by the two methods. For example, for the 2-D Otsu method, there is only one image to be well segmented as shown in Fig. 10d, and for the 2-D maximum entropy method, there are two images to be well segmented as shown in Figs. 8e and 9e, respectively. In comparison with these three methods, the visual thresholding results by the proposed method indicate that, in addition to a completely segmenting for the targets from complex background, the new method exhibits less background noises. This could be attributed to the utilization of the background constraint for the original image. The background constraint dramatically simplifies the original infrared image by weakening the gray level changes of background. In addition, one can observe that the segmented results of the new method are comparative with those by manual segmentation in visual effect. As a whole, from the visual segmented results it can be concluded that the proposed method enjoys better visual effect, indicating the new method with better segmentation performance.

### Segmentation accuracies comparison of different methods

To quantitatively compare the segmentation accuracy of the proposed method with other thresholding methods, the threshold of manual segmentation is regarded as the optimal threshold, and the threshold accuracy can be defined as

17 $$A_{th} = \left( {1 - \frac{{\left| {T - T_{o} } \right|}}{{T_{o} }}} \right) \times 100\,\% ,$$

where $T$ is the threshold obtained by thresholding method and $T_{o}$ denotes the optimal threshold.

The threshold accuracy defined by Eq. (17) describes the approximation degree of the threshold given by the thresholding method to the optimal threshold of an image, which can reflect the segmentation accuracy of thresholding method. Obviously, the higher the threshold accuracy is, the better the segmentation accuracy. Hence, the threshold accuracy could represent the segmentation accuracy of the thresholding method to some extent. Table 1 lists the thresholds and their accuracies of different methods. Because the threshold obtained by the 2-D histogram based method is of two dimensions, the mean of the 2-D threshold is used for the threshold accuracy calculation. The data show that, during the four threshoding methods, the threshold accuracy of the standard Otsu method is the lowest with the mean of only 74.7 %, and the one of the proposed method is the highest with the mean of 97.1 % much bigger than the second highest 82.3 % of 2-D maximum entropy method. In addition, all the threshold accuracies of the proposed method are not smaller than 94.2 % and the maximum of them even reaches to 100 %, implying better robustness of the new method. So from the quantitative analysis in this section, one can conclude that the proposed method has higher segmentation accuracy and is with better robustness.

**Table 1:** Thresholds and accuracies of different thresholding methods

Images	Optimal threshold	Otsu method		2-D Otsu method		2-D maximum entropy		This paper method	
		Threshold	*A*_*th*_ (%)	Threshold	*A*_*th*_ (%)	Threshold	*A*_*th*_ (%)	Threshold	*A*_*th*_ (%)
Figure 5a	138	84	60.9	(84,85)	61.2	(101,108)	75.7	138	100.0
Figure 6a	140	115	82.1	(115,131)	87.9	(118,118)	84.3	145	96.4
Figure 7a	100	79	79	(80,80)	80.0	(122,105)	86.5	99	99.0
Figure 8a	135	104	77.0	(104,105)	77.4	(138,138)	97.8	137	98.5
Figure 9a	125	105	84.0	(105,106)	84.4	(123,123)	98.4	131	95.2
Figure 10a	121	115	95.0	(115,116)	95.5	(153,153)	73.6	126	95.9
Figure 11a	155	85	54.8	(85,84)	54.5	(112,112)	72.3	146	94.2
Figure 12a	143	92	64.3	(92,94)	65.0	(97,103)	69.9	139	97.2
Average	–	–	74.7	–	75.7	–	82.3	–	97.1

### Running time comparison of different methods

Table 2 lists the running times of different methods. The data show that the standard Otsu method consumes the least running time in these methods with mean of only 1.89 ms, however, the proposed method wastes only slightly more time than that by the standard Otsu method with mean of 9.22 ms, implying its good performance in real time processing. The new method reduces search space during thresholding from the whole gray levels of an original image to a much smaller range [*T*_*c*_, *L* − 1] and hence saving running time. Nevertheless, the new method needs extra time to estimate the ranges of target and background, with implementing background constraint. In addition, one can observe that the two methods based on 2-dimensional histogram cost too much more times with three orders of magnitude higher than both the standard Otsu method and the proposed method. This is because that the utilization of two-dimension histogram makes that the complexity of the algorithm increases exponentially as gray levels of image. In a word, one can conclude that the proposed method consumes less running time in segmentation of an image, implying its better performance in real time processing.

**Table 2:** Running times of different thresholding methods

Methods	Figure 5a	Figure 6a	Figure 7a	Figure 8a	Figure 9a	Figure 10a	Figure 11a	Figure 12a	Average
Standard Otsu method (ms)	1.45	1.87	1.49	1.63	1.6	3.45	1.88	1.76	1.89
2-D Otsu method (ms)	1459.18	1393.91	1534.09	1358.98	1391.35	2573.63	1520.35	1478.4	1588.74
2-D maximum entropy (ms)	4348.39	4361.48	4504.75	4030.64	4287.01	8039.61	4430.92	4411.26	4801.76
This paper method (ms)	9.372	9.914	6.79	8.888	8.868	11.346	8.834	9.743	9.22

## Conclusions

In this paper, the threshold of the Otsu method is analyzed and the underlying reason of its failure in segmentation of infrared images in complex background is presented. It is concluded that the extremely large difference of within-class variances between background and target regions leads to deviation of threshold given by Otsu method from the optimal. From this conclusion, an unsupervised background constrained method is proposed for segmentation of infrared images in complex background based on the Otsu method. Due to the complexity of background, the original infrared image is divided into three classes of target region, middle background and lower background based on the statistical characteristics of original image, firstly. Then, the background constraint is implemented based on the within-class variance of target region and hence the original image can be simplified. Finally, the Otsu method is applied to simplified image for threshold selection. Experimental results on a variety of tank infrared images in complex background demonstrate that the proposed method has better segmentation performance and even can be comparative with the manual segmentation. In addition, its average running time is only 9.22 ms, implying the new method with good performance in real time processing.
